# Upcycling Waste Polyethylene Terephthalate to Produce Nitrogen-Doped Porous Carbon for Enhanced Capacitive Deionization

**DOI:** 10.3390/molecules29204934

**Published:** 2024-10-18

**Authors:** Hui Yu, Haiyan Duan, Liang Chen, Weihua Zhu, Daria Baranowska, Yumeng Hua, Dengsong Zhang, Xuecheng Chen

**Affiliations:** 1 College of Science, Beihua University, Jilin City 132013, China; huiyu19780413@163.com (H.Y.); zhuwhcc@126.com (W.Z.); 2 School of Materials Science and Engineering and Research Center of Nano Science and Technology, Shanghai University, Baoshan, Shanghai 200444, China; hyduan1020@126.com; 3 Soybean Research Institute, Jilin Academy of Agricultural Sciences, Changchun 130033, China; liangchen119@126.com; 4 Faculty of Chemical Technology and Engineering, West Pomeranian University of Technology, 71-065 Szczecin, Poland; daria_baranowska@zut.edu.pl (D.B.); yumeng.hua@zut.edu.pl (Y.H.)

**Keywords:** PET, MOF, N-PC, CDI

## Abstract

Porous carbon with a high surface area and controllable pore size is needed for energy storage. It is still a significant challenge to produce porous carbon in an economical way. Nitrogen-doped porous carbon (N-PC) was prepared through carbonization of a mixture of waste PET-derived metal–organic frameworks (MOFs) and ammonium. The obtained N-PC exhibits a large surface area and controlled pore size. When utilized as an electrode material for supercapacitors, the N-PC exhibits a specific capacitance of 224 F g^−1^, significantly surpassing that of commercial activated carbon (AC), which has a capacitance of 111 F g^−1^. In the subsequent capacitive deionization (CDI) tests, the N-PC demonstrated a maximum salt adsorption capacity of 19.9 mg g^−1^ at 1.2 V in a NaCl electrolyte (0.5 g L^−1^), and the salt adsorption capacity increased to 24.7 mg g^−1^ at 1.4 V. The N-PC electrode also exhibited superior regeneration. The present work not only presents a potential approach to develop cost-effective electrodes for seawater purification but also paves the way for recycling of waste plastics into high value-added products.

## 1. Introduction

As the majority of water exists in the form of seawater, desalination plays a crucial role in generating fresh water to meet the ever-increasing demand of the future global population. Compared with other desalination techniques that require high energy and complex operations [1,2,3], capacitive deionization (CDI) is considered a novel technology due to its energy efficiency, environmental friendliness, and cost-effectiveness [4,5,6]. CDI purifies water by adsorbing ions onto the electrode surface to form electric double layers (EDLs) [7] during the electrochemical process, a mechanism derived from electric double-layer capacitors (EDLCs). Consequently, the electrical conductivity and structural properties of the electrode materials significantly influence CDI performance. Although electrically conductive carbonaceous materials with high porosity and large specific surface areas have been proposed as CDI electrode materials with high salt adsorption capacity (SAC) [8,9,10,11,12], further improvements are necessary to meet commercial demands, particularly concerning the mesopore-to-micropore ratio and the doping of other elements.

It has been demonstrated that materials with optimal mesopore-to-micropore ratios can enhance the electro-sorption capacity of ions by providing additional pathways and storage capacity [13,14,15,16]. In particular, nitrogen doping positively impacts capacitive deionization (CDI) by improving electrical conductivity and wettability, as well as facilitating salt adsorption through Faradaic reactions [17,18,19,20,21]. This is due to the doping of nitrogen, which will enhance the pseudocapacitance of the capacitor, leading to increased salt adsorption capacity. Han et al. elucidated the relationship between the pore size of carbon materials and CDI performance [13]. Although the mechanisms of salt adsorption in micropores and mesopores differ, both types of pores in carbon materials primarily contribute to Na^+^ removal in CDI. Xu et al. proposed mechanisms to elucidate the positive effects of nitrogen species on CDI. It is believed that during CDI, Na^+^ ions interact with pyridinic nitrogen through metal–ligand bonds, thereby promoting electrosorption [22]. Consequently, the presence of mesopores and nitrogen doping can enhance CDI properties.

On the other hand, metal–organic frameworks (MOFs) are among the most promising materials for synthesizing porous carbon due to their controllable pore sizes and flexible structures [23,24,25,26,27]. However, the high cost of MOFs significantly hinders their widespread application across various fields. To address this challenge, we present a highly efficient method for recycling waste PET into MOFs with a high yield. Because of the low cost of waste PET, it is cost-effective to produce MOFs [28]. At the same time, the use of PET as a carbon source for the production of MOFs will also alleviate the environmental pressure caused by waste plastic. Following this process, an interconnected, nitrogen-doped porous carbon (N-PC) was produced on a large scale by carbonizing a mixture of MOFs and urea. The resulting N-PC exhibits a high surface area and an optimal meso-to-micropore ratio, making it a promising electrode material for CDI.

## 2. Results

**Figure 1:**
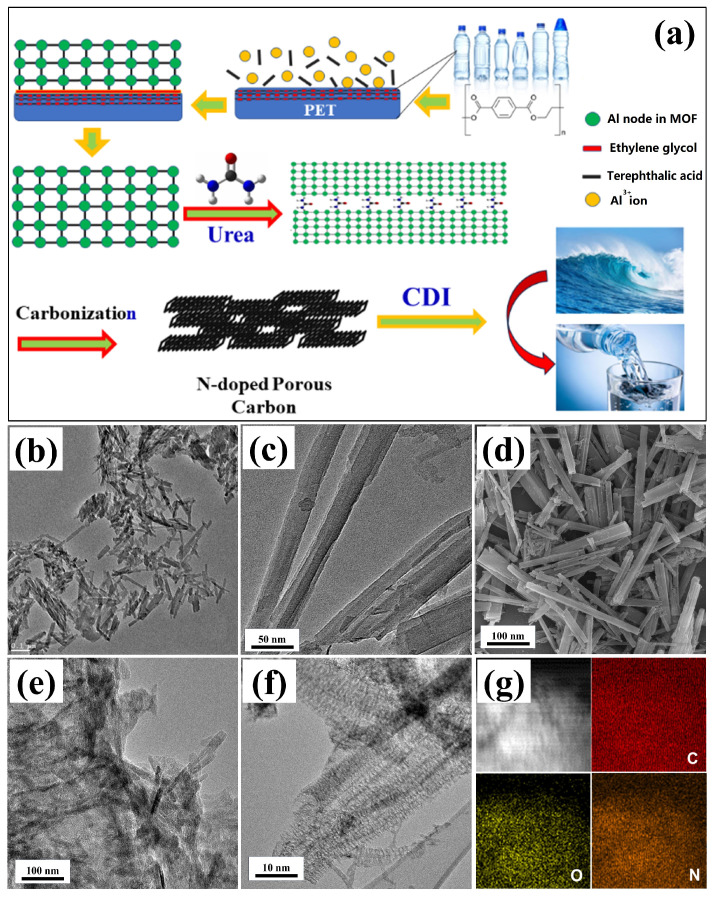
(**a**) Schematic illustration of the synthesis of N-PC from waste PET and application in desalination. (**b**,**c**) TEM and (**d**) SEM images of waste PET-derived MOF-Al. (**e**) TEM image of the mixture of PET-derived MOF-Al and urea and (**f**) N-PC. (**g**) STEM image and elemental maps for carbon, oxygen, and nitrogen in the N-PC.

**Figure 2:**
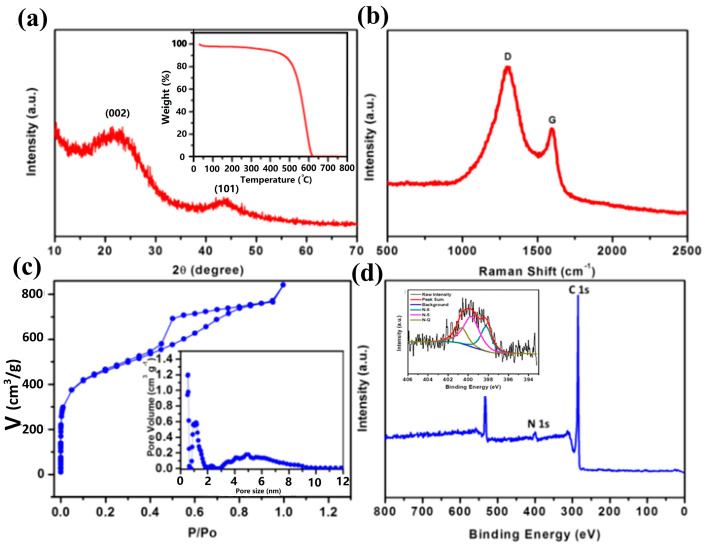
(**a**) XRD pattern (inset: TGA curve), (**b**) Raman scattering spectrum, (**c**) N_2_ adsorption/desorption isotherms (inset: pore size distribution), (**d**) XPS survey spectrum of the N-PC.

As depicted in Figure 1a, the interconnected N-PC materials are synthesized by annealing the mixture of PET-derived MOF-Al/urea at elevated temperatures. The resulting PET-derived MOF-Al crystals exhibit a needle-like morphology with diameters of 20–50 nm and lengths of 100–200 nm, along with a porous structure (Figure 1b,c). Upon mixing with urea, the MOF-Al crystals aggregate to form a larger precursor through reaction with the positive amine groups (Figure 1e). Subsequently, a 3D carbon network with an interconnected structure is formed from this precursor via high-temperature annealing. The interconnected N-PC has a micrometer-scale size and exhibits a porous structure observed using TEM (Figure 1f). The TEM image further reveals the presence of numerous micropores and mesopores within the agglomerated N-PC, which are conducive to its excellent capacitive performance. To investigate the reaction between MOF-Al and urea, STEM and EDS analyses were also conducted (Figure 1g). The results show monodispersed N and overlapping distributions of C, O, and N, confirming the successful nitrogen doping from urea. Given these advantages, the N-PC with high porosity is utilized for desalination (Figure 1a). Figure 2a presents the XRD pattern, which exhibits two distinct diffraction peaks characteristic of carbon, along with a left shift in the (002) peak and a broadening of the (101) peak, indicative of the low crystallinity of the N-PC. In Figure 2b, the I_G_/I_D_ ratio derived from MOFs is less than 1, suggesting a disordered N-PC structure due to the porous nature and potential elemental doping. XRD and Raman data further suggest that the PET-derived N-PC is a promising candidate for CDI applications.

The interconnected N-PC’s porous characteristics were elucidated through N_2_ adsorption/desorption analysis (Figure 2c). The type IV isotherm profile suggests the presence of micro-, meso-, and macropores within the N-PC. Significant adsorption/desorption at a low pressure indicates the presence of micropores and the presence of mesopores at ~5 nm, as confirmed by the pore size distribution (inset in Figure 2c). Mesopores not only enhance ion diffusion but also enhance the storage of the salt solution during CDI [13]. Figure 2c indicates that the N-PC has a specific surface area of 1606 m^2^ g^−1^ and a pore volume of 1.3 cm^3^ g^−1^. The TGA analysis (inset in Figure 2a) revealed that no weight remained after heating in air at 800 °C, suggesting the complete removal of metal components from the interconnected N-PC. XPS analysis was conducted to characterize the surface chemistry of the interconnected N-PC (Figure 2d), revealing the presence of carbon, oxygen, and a small amount of nitrogen (2.9%). The XPS spectrum of the N-PC shows it contains carbon, oxygen, and nitrogen. In the inset figure of Figure 2d, the N1s peak of N-PC is de-convoluted into three characteristic peaks located at 398.2, 399.7, and 400.6 eV, which are assigned to pyridinic nitrogen (N-6), pyrrolic nitrogen (N-5), and quaternary nitrogen, also known as graphitic nitrogen (N-Q), respectively. The nitrogen in the N-PC is composed of 28.9% N-6, 51.5% N-5, and 19.6% N-Q. This composition is advantageous for enhancing wettability and electrical conductivity [18]. Furthermore, the additional pseudocapacitance resulting from nitrogen doping significantly improves the CDI performance [17].

**Figure 3:**
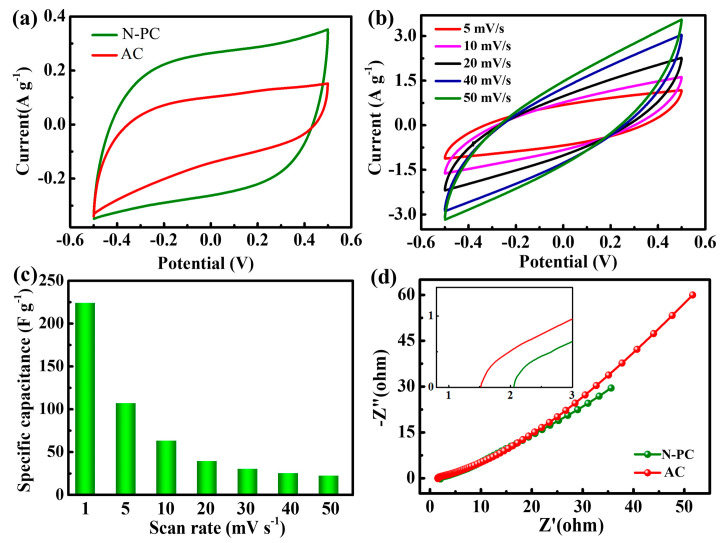
(**a**) Cyclic voltammetry (CV) curves of AC and N-PC at a scan rate of 1 mV s^−1^; (**b**) CV curves of the N-PC electrode over a scanning rate range of 5 to 50 mV/s; (**c**) specific capacitances of the N-PC determined from CV curves at various scanning rates; (**d**) electrochemical impedance spectroscopy (EIS) profiles of N-PC and AC, depicted as Nyquist plots, with the inset illustrating the magnified high-frequency region. The data were collected from a 0.5 M aqueous NaCl solution.

Figure 3a illustrates the cyclic voltammetry (CV) curves of the N-PC and the commercial activated carbon (AC) electrodes at a scan rate of 1 mV s^−1^, spanning from −0.5 to 0.5 V. Both electrodes display quasi-rectangular shapes. The current gradually increases and then decreases within the selected potential range, indicating that salt ions are electro-sorbed through coulombic interactions and faradaic reactions [29,30]. A greater area within the CV curve corresponds to a higher specific capacitance. In this regard, the N-PC electrode exhibits a larger CV area than the AC electrodes, suggesting enhanced specific capacitance and electro-sorption capabilities, which is because N-PC has more open pores or active sites than AC. The specific capacitance of the N-PC is determined to be 224 F g^−1^, which is notably higher than that of AC (111 F g^−1^), suggesting that N-PC enhances the propagation of salt ions. The N-PC exhibits rectangular cyclic voltammetry (CV) curves across a range of scanning rates from 5 to 50 mV s^−1^ in a 0.5 M NaCl solution.

As depicted in Figure 3b, all CV curves exhibit symmetry about the X-axis, confirming the reversibility of the capacitive properties and the stable rectangular shape, which indicates exceptional electrochemical capacitive behavior. The specific capacitances derived from the CV curves at various scanning rates are presented in Figure 3c. The specific capacitance of the N-PC electrode decreases as the scanning rate increases from 1 to 50 mV s^−1^. This can be attributed to the following: Initially, at lower scanning rates, there is ample time for ions to migrate into the electrode interior, leading to higher electro-sorption of ions and improved capacitive performance. However, at high scanning rates, ions have insufficient time to diffuse into the deeper pores, resulting in a reduced accessible surface area for ion adsorption and a decrease in specific capacitance [31]. Additionally, the formation of the double electric layer is affected by the increased resistance to ion diffusion within the pores at high scanning rates [32]. Figure 3d shows the Nyquist plots of the N-PC and AC electrodes in a 0.5 M aqueous NaCl solution, which exhibit a similar shape with a linear region at low frequencies and a small semicircle at high frequencies [33]. The straight and inclined lines of both electrodes at low frequencies confirm electrostatic adsorption and capacitive behavior. At high frequencies, the small semicircle is parallel to the polarization resistance, which includes the charge transfer resistance between the electrode surface and the solution [34]. The charge transfer resistance for both the N-PC and AC electrodes is negligible. Moreover, the point where the semicircle intersects the real axis corresponds to the equivalent series resistance (ESR) of the electrodes, which is attributed to contact resistance between the active materials and current collectors, ionic resistance of the salt solution, and the intrinsic resistance of the electrodes. Generally, the ESR value reflects the internal resistance of the electrodes under similar measurement conditions [35]. It is evident that the ESR of the PC electrode is lower than that of the AC electrode, suggesting that N-PC exhibits higher conductivity. In the inset figure of Figure 3d, the ionic resistance of AC is lower than N-PC, but the series resistance of the AC cell is larger than the N-PC [34].

**Figure 4:**
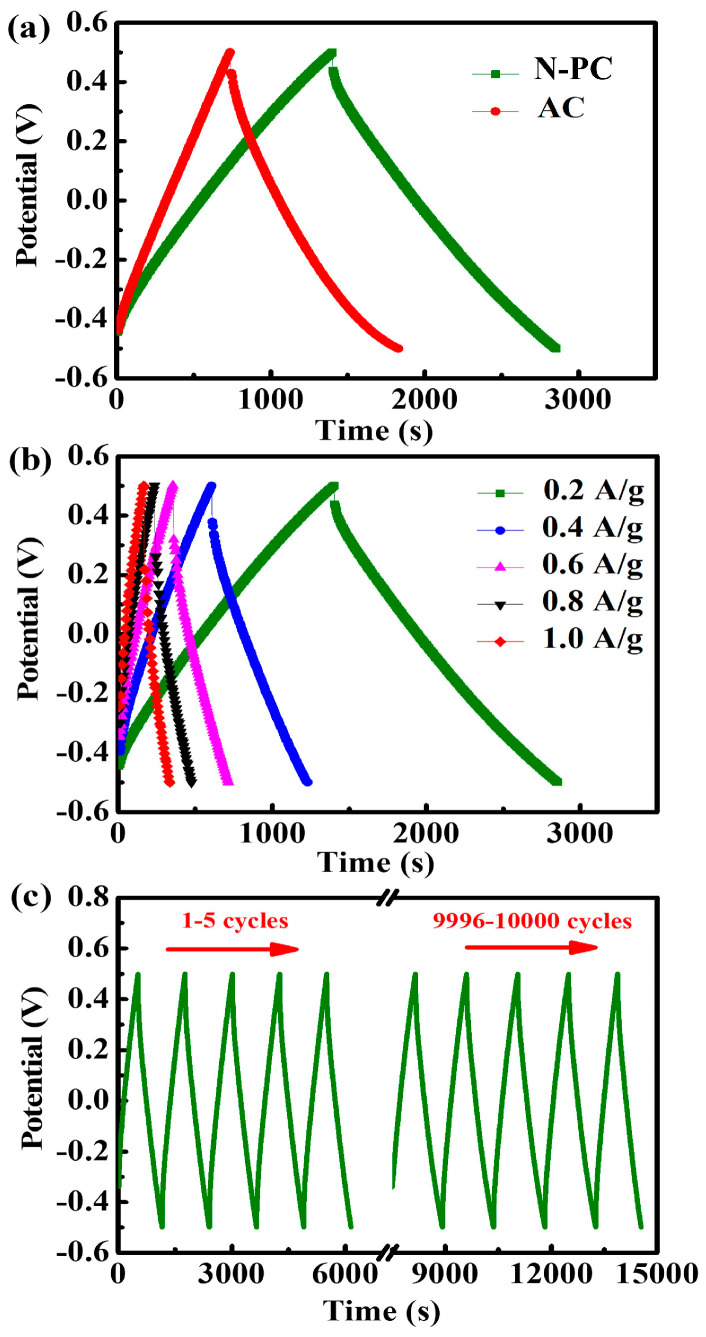
(**a**) Charge/discharge curves at a current density of 0.2 A g^−1^; (**b**) charge/discharge curves across a range of current densities from 0.2 to 1 A g^−1^; (**c**) cycle performance of the N-PC electrode at a current density of 0.4 A g^−1^ (showing the first and last five cycles). All tests were conducted in a 0.5 M NaCl solution.

GCD tests were conducted to investigate electro-sorption within the potential range of −0.5 V to 0.5 V at a current density of 0.2 A g^−1^. As depicted in Figure 4a, the GCD curves for the N-PC and AC electrodes display a triangular shape within the selected potential range, indicating superior reversibility. The linear and symmetrical charge–discharge profiles confirm the excellent I–V response and electrochemical double-layer capacitor (EDLC) behavior [36], consistent with the cyclic voltammetry results. The discharge time of the N-PC electrode is much longer than that of the AC electrode, suggesting a higher specific capacitance for the N-PC. The abrupt voltage drop (iR drop) at the beginning of the discharge process reflects the internal resistance of the electrodes [37]. Here, the iR drop of the N-PC electrode is notably smaller than that of AC, further confirming that the N-PC material exhibits low resistance due to its interconnected structure. Overall, the N-PC electrode demonstrates lower internal resistance and higher conductivity, making it promising for CDI applications with reduced energy consumption. Figure 4b presents the charge–discharge profiles of the N-PC electrode at various current densities from 0.2 to 1.0 A g^−1^. The discharge time decreased with an increase in current densities, which is due to the use of only the surface layer of porous carbon, leading to the discharge time being decreased. The profiles maintain a triangular shape even at elevated current densities, indicating superior reversibility and capacitive behavior [38]. The cycling performance is a critical factor influencing the stability of electrodes. The continuous GCD curves of the N-PC electrode at 0.4 A g^−1^ are depicted in Figure 4c. After 10,000 cycles, the curves in the final five cycles maintain the characteristic triangular shape, akin to the initial five cycles, confirming its excellent stability. Moreover, no discernible charge–discharge decay is observed during repeated cycling, thereby substantiating the superior cycling performance of the N-PC electrode.

**Figure 5:**
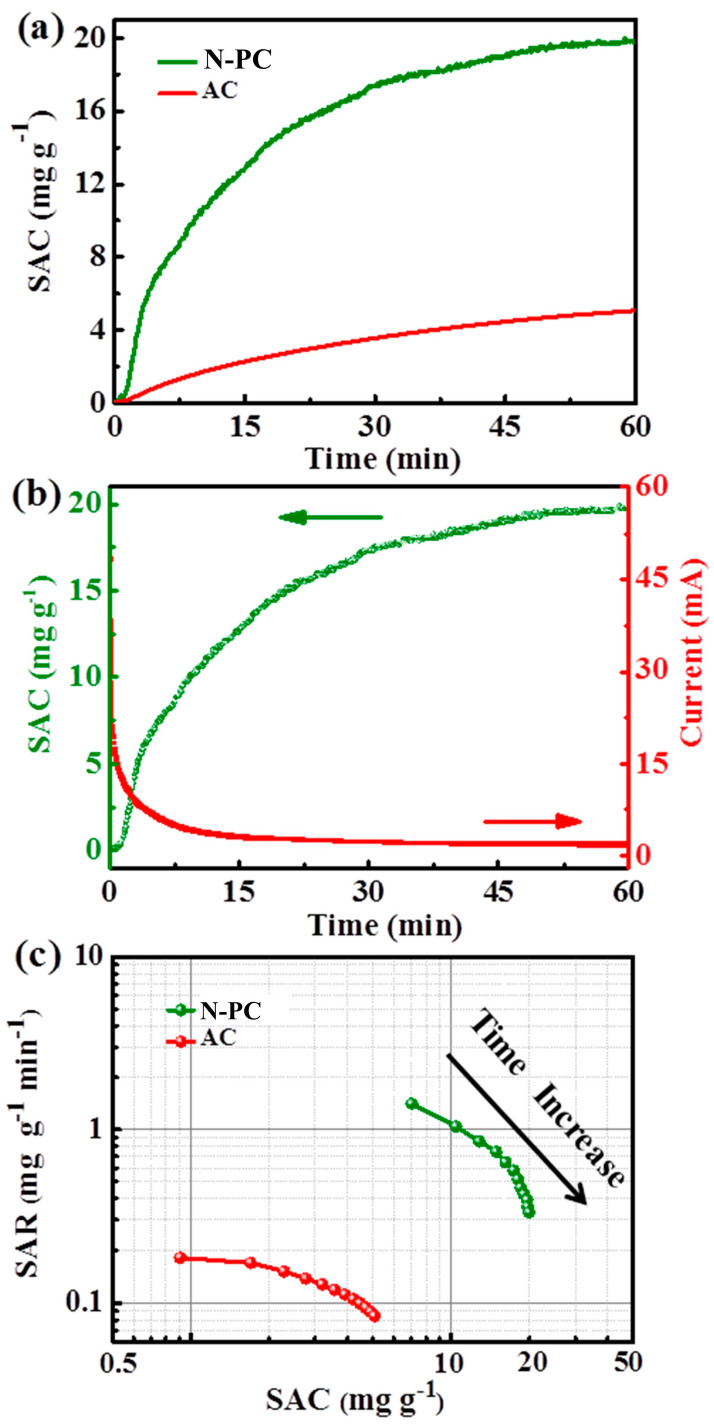
(**a**) SAC vs. deionization time for the N-PC and AC electrodes, (**b**) current vs. time and SAC vs. time, and (**c**) Ragone plots (SAR vs. SAC) for the N-PC electrode in a 500 mg L^−1^ NaCl solution at 1.2 V.

The CDI performance of the N-PC and commercial AC electrodes was initially evaluated under conditions of 500 mg L^−1^ NaCl and 1.2 V. The plots of SAC versus time are presented in Figure 5a. Both electrodes exhibit a significant enhancement in SAC during the initial stage as the voltage increases. This is attributed to the ample surface area available for ion adsorption and the weak electrostatic repulsion. As the deionization time is extended, the SAC initially increases and then stabilizes, reaching electro-sorption equilibrium after 1 h. The rate of SAC increase is consistently higher for the N-PC electrode compared to AC, suggesting that ions adsorb more readily onto the former. The N-PC exhibits a higher SAC (19.9 mg g^−1^) than AC (5.1 mg g^−1^) due to its physical properties derived from the MOF-derived porous structure during CDI.

Furthermore, the N-PC exhibits a higher SAC than previously reported results (Table 1). For the N-PC, a low charge efficiency of 0.49 indicates reduced energy consumption during the CDI test (Figure 5b). Weak adhesion between the active materials and graphite paper, the rejection of co-ions, and the blocking effects of the binder used may account for the efficiency values below 1.0. To change the supporting substrate, tuning the pore structure of porous carbon and avoiding the use of a binder will further improve the efficiency. Considering the SAC and SAR, the Ragone plots for N-PC and AC are presented in Figure 5c. These graphs illustrate the overall CDI performance by plotting SAR against SAC [39]. The SAR of the electrodes decreases over time during deionization until reaching adsorption equilibrium, suggesting a reduction in effective surface area and increased electrostatic repulsion with extended deionization time. The N-PC electrode exhibits shifts towards the upper and right regions, indicating higher SAR and SAC. Based on the above analyses, the unique 3D carbon network, which endows the N-PC with a large accessible surface area and low internal resistance, contributes to its enhanced CDI performance.

**Figure 6:**
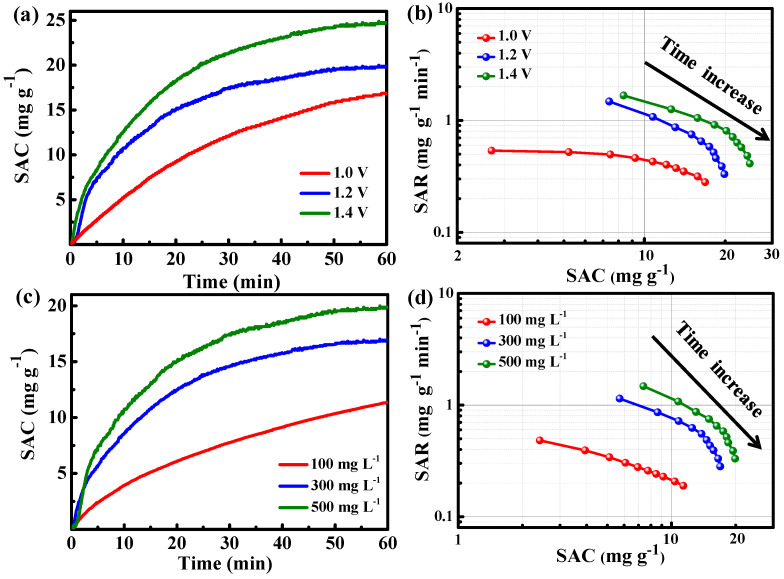
(**a**) Graphs depicting the relationship between SAC and deionization time, and (**b**) Ragone plots illustrating the specific areal rate (SAR) versus SAC for N-PC electrodes at varying NaCl concentrations; (**c**) Graphs showing the SAC versus deionization time, and (**d**) Ragone plots depicting the SAR versus SAC for N-PC electrodes at different voltages.

Figure 6a,b present graphs of deionization time versus SAC in a 500 mg L^−1^ NaCl electrolyte solution using N-PC at voltages ranging from 1.0 to 1.4 V. The SAC of all the curves increases upon application of voltage, reaching equilibrium within one hour. At higher voltages, the SAC is significantly enhanced, indicating that more Na^+^ ions can be adsorbed at larger voltages. The SAC increases from 16.9 to 24.7 mg g^−1^ within the voltage window of 1.0 to 1.4 V. Additionally, the Ragone plot in Figure 6b demonstrates shifts towards the upper and right regions as the voltage increases from 1.0 to 1.4 V, indicating higher SAR and SAC. The enhanced coulombic forces between electrodes and ions at elevated voltages facilitate faster and more efficient adsorption. No bubbles are observed at voltages as high as 1.4 V due to the voltage drop caused by circuit resistance [40,41].

The impact of salt concentrations (100, 300, and 500 mg L^−1^) on the performance of CDI was also examined. As illustrated in Figure 6c,d, the SAC increases from 11.4 to 19.9 mg g^−1^ as the electrolyte concentration is increased (from 100 mg L^−1^ to 500 mg L^−1^). Figure 6d presents the corresponding Ragone plots, which shift to the upper and right quadrants with higher salt concentrations, indicating enhanced specific areal rates (SAR) and increased SAC. In concentrated electrolytes, the formation of a compact electrochemical double layer (EDL) contributes to the increased SAC, while improved conductivity accelerates ionic transport, resulting in a faster SAR [42,43,44]. As shown in Table 1, our work is super to most of the previously published results.

**Table 1:** Electrosorption capacities of nitrogen-doped carbon electrodes including our N-PC samples.

Entry	Component	NaCl Solution (mg/L)	Applied Voltage (V)	SAC (mg/g)	Ref.
1	NHCF-800	500	1.2	30.1	[45]
2	N,B-NPC	500	1.2	21.5	[46]
3	A-HCMs	400	1.0	14.64	[47]
4	3D-NPC	500	1.34	19.4	[48]
5	NPC	500	1.2	19.5	[49]
6	N-porous carbon	500	1.2	14.63	[50]
7	N-porous carbon	500	1.2	12.56	[51]
8	N-ALS	500	1.2	24.79	[52]
9	C(ZIF-8)	58.44	1.2	8.52	[53]
10	F-N-GPM	100	1.8	21.8	[54]
11	N-PC	500	1.2/1.4	19.9/24.7	This work

**Figure 7:**
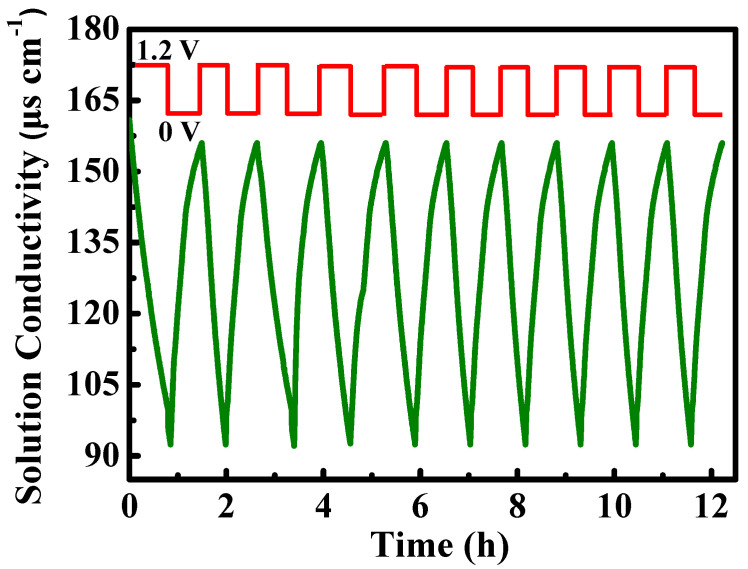
Cycling deionization–regeneration curves for PET-based N-PC (NaCl concentration: 100 mg L^−1^, voltage: 1.2 V).

Figure 7 illustrates the performance of the N-PC electrodes over 10 cycles, demonstrating excellent repeatability and regeneration capabilities. To evaluate the cycling performance of the CDI process, adsorption/desorption experiments were conducted at a NaCl concentration of 100 mg L^−1^ and a voltage of 1.2 V. A short circuit (0 V) was employed for both electrodes to desorb ions and return them to the solution. The second adsorption process commenced once the conductivity reverted to its initial value from the first CDI cycle.

## 3. Materials and Methods

PET bottles were obtained from commercial soft drink packages, aluminum nitrate nonahydrate (Al(NO_3_)_3_·9H_2_O), N,Ndimethylformamide (DMF), urea, and hydrochloric acid (HCl) were bought from Chempur, Piekary Śląskie, Poland. The commercial activated carbon (AC, Vulcan XC 72) was purchased from Sigma-Aldrich (St. Louis, MO, USA). In this experiment, all the chemicals were used directly without any further purification, and deionized water was used throughout the experiments. Software: Office 2010, Origin 2023, CorelDRAW 2019. 

### 3.1. Preparation of N-PC Materials

To produce N-PC, aluminum-based metal–organic frameworks (MOF-Al) were first prepared from waste PET according to previously described procedures [28]. Subsequently, in a typical synthesis, 0.4 g of urea was mixed with 1 g of PET-derived MOF-Al in DMF. After stirring for 12 h, the mixture of MOF-Al and urea was washed, dried, and heated to 850 °C in an argon atmosphere for 3 h. The product was then refluxed in hydrochloric acid (HCl) solution to remove Al_2_O_3_, filtered, washed with deionized water, and placed in a vacuum dryer at 90 °C. Finally, N-PC was obtained from the above process for further use.

### 3.2. Characterization

X-ray diffraction (XRD, X’Pert PRO Philips diffractometer, Co Kα radiation) and transmission electron microscopy (TEM, FEI Tecnai F20, FEI Company, Hillsboro, OR, USA) were employed to examine the microstructure of the produced N-PC. Raman spectra were acquired using an InVia Raman microscope (Renishaw, Hong Kong, China) using an excitation wavelength of 785 nm. Scanning transmission electron microscopy (STEM) and energy-dispersive X-ray spectroscopy (EDS) were utilized to determine the elemental composition, while nitrogen adsorption/desorption isotherms (Micromeritics, Norcross, GA, USA) were obtained to assess the porous properties. Additionally, X-ray photoelectron spectroscopy (XPS, Prevac, Rogów, Poland) was used. The measurements were conducted using Mg Kα (hν = 1253.6 eV) radiation in a PREVAC (Rogów, Poland) system equipped with a Scienta SES 2002 (ScientaOmicron, Uppsala, Sweden) electron energy analyzer operating with a constant transmission energy (Ep = 50 eV). Thermogravimetric analyses (TGA, DTA-Q600, TA Instruments, New Castle, DE, USA) were conducted to evaluate the chemical composition and related properties.

### 3.3. CDI Tests

To assess the electrochemical and CDI properties, a blend of N-PC and polytetrafluoroethylene (PTFE) with a mass ratio of 9:1 was prepared in acetone. This mixture was then used to create a carbon slurry, which was subsequently coated onto carbon paper and heat-treated at 120 °C for 8 h to fabricate the working electrode. Electrochemical impedance spectroscopy (EIS) and cyclic voltammetry (CV) measurements were conducted using an electrochemical workstation (CHI 660D, ArtisanTG, Champaign, IL, USA). Galvanostatic charge–discharge (GCD) tests were performed using an automatic LAND battery testing instrument (Landt Instruments, Vestal, NY, USA). The electrochemical experiments were conducted in a three-electrode system, utilizing a 0.5 M NaCl electrolyte solution. The initial NaCl concentrations were 100, 300, and 500 mg/L. The cell voltages were 0.8, 1.0, 1.2, and 1.4 V. Graphite served as the counter electrode, while a saturated calomel electrode functioned as the reference electrode. The specific capacitance (C) was calculated using the following equation:

(1) C=(∫IdV)/2vm∆V

Here, C denotes the specific capacitance, I represents the current density, m is the mass of the active material, ν is the scanning rate, and ΔV is the potential window.

CDI experiments were conducted in a recycling cell equipped with two-sided electrodes. The total active mass was 160 mg, and the dimensions of the electrodes were 60 mm × 70 mm × 0.2 mm. A grid spacer with a thickness of 0.27 mm was employed as a separator. In the experiment, 35 mL of NaCl solution was introduced into the cell at a flow rate of 40 mL min^−1^ using a pump. The NaCl concentrations in the solutions were 100, 300, and 500 mg L^−1^, with corresponding voltages of 1.0, 1.2, and 1.4 V. During the CDI test, the NaCl concentrations were continuously monitored and measured at the cell outlet using a conductivity meter. The SAC was calculated using the following equation:

(2) $$SAC=\left(C_{0}-C\right)V/m$$

where C_0_ and C are the initial and final concentrations of NaCl in the solutions, m is the total mass of the active material, and V is the total volume of the NaCl solution. The salt adsorption rate (SAR) was calculated using the following equation:

(3) SAR=SAC/t

where SAC denotes the salt adsorption capacity and t represents the deionization time. The charge efficiency (Λ) indicates the electric double layer formed at the interface between the electrode and solution, and it is expressed as the ratio of the amount of removed salt ions to the electrical charge, detailed as follows:

(4) :Λ=F×Γ/∑

where F is the Faraday constant (96,485 C mol^−1^), Γ is the salt adsorption capacity (mol g^−1^), and Σ (charge, C g^−1^) is determined by integrating the corresponding current.

## 4. Conclusions

PET-derived MOF was employed as a carbon source for the synthesis of N-PC. The resulting PET-derived N-PC exhibited a specific capacitance of 224 F g^−1^ and a maximum SAC of 19.9 mg g^−1^, which was notably higher than commercial AC. This good performance is attributed to its rapid ion diffusion facilitated by micropores and mesopores, as well as contributions from the Faradaic reaction. The SAC of the N-PC increased with voltage, indicating strong coulombic interactions between the electrode and ions at high voltages. The high SAC, excellent regeneration properties, and overall performance confirm the potential of PET-derived N-PC as a cost-effective electrode material for CDI. Additionally, this research proposes a straightforward method for upcycling PET waste into valuable products. Finally, N-PC from waste PET can be potentially applied in energy storage and polluted water treatment.

## Data Availability

The original contributions presented in the study are included in the article, further inquiries can be directed to the corresponding authors.
